# The Transition Care Index: Standardizing comprehensive transition and transfer for young adults with inflammatory bowel disease

**DOI:** 10.1002/jpr3.70045

**Published:** 2025-06-17

**Authors:** Hilary K. Michel, Jennifer L. Dotson, Jennie G. David, Amy Donegan, Ashley Kiel, Ross M. Maltz, Hannah McKillop, Melanie Oates, Brendan Boyle

**Affiliations:** ^1^ Department of Pediatrics The Ohio State University College of Medicine Columbus Ohio USA; ^2^ Division of Gastroenterology, Hepatology, and Nutrition Nationwide Children's Hospital Columbus Ohio USA; ^3^ Center for Child Health Equity and Outcomes Research The Research Institute, Nationwide Children's Hospital Columbus Ohio USA; ^4^ Pediatric Gastroenterology Arkansas Children's Hospital/University of Arkansas for Medical Sciences Little Rock Arkansas USA; ^5^ Pediatric Psychology and Neuropsychology Nationwide Children's Hospital Columbus Ohio USA

**Keywords:** adolescent, Crohn's disease, gastroenterology, quality improvement, ulcerative colitis

## Abstract

**Objectives:**

In young adults with inflammatory bowel disease (IBD), the time following transfer to adult care is high‐risk for adverse outcomes. We used quality improvement (QI) methods to standardize care, decrease variation, and improve preparation of young adults during the transition/transfer process.

**Methods:**

We created the IBD Transition Care Index (TCI), a list of 10 variables whose completion was felt to represent a more comprehensive transition/transfer process. Variables were organized into three domains: Disease Control/Physical Health, Psychosocial Well‐being, and Transition/Transfer Preparation. We educated patients, caregivers, and providers on the value of completing the TCI to deliver complete, multidisciplinary preparation. We recorded variable completion in a database, reviewed results regularly with providers, and compared rates of variable completion between IBD‐focused and general gastroenterology (GI) physicians.

**Results:**

Three hundred twenty‐two patients transferred to adult care during the project period (211 pre‐intervention and 121 post‐intervention). In the overall cohort, the mean percentage of TCI variables completed increased from a baseline of 62%–71% in the post‐intervention period, with a significant increase in the rate of multidisciplinary IBD annual visit (IBD AV) attendance (51% vs. 62%, *p* = 0.03). Patients cared for by general GI physicians had significantly increased rates of both overall TCI variable completion (54% vs. 72%, *p* = 0.02) and IBD AV attendance (34% vs. 57%, *p* = 0.02) in the pre‐ versus post‐intervention period.

**Conclusions:**

Care Indexes such as the TCI can be used to reduce variability and standardize complex clinical processes like transition/transfer for young adults with IBD, with the goal of improving patient outcomes.

## Abstract (highlights)

**What is Known:**

- Transition and transfer are inevitable processes for adolescents and young adults (AYAs) with chronic diseases like inflammatory bowel disease (IBD), yet preparation for transition is variable and often, absent.
- Patients with IBD are known to be at risk for poor adherence, increased disease activity, and need for unplanned care in the months following transfer to adult IBD care.

**What is New:**

- Using the IBD Transition Care Index, we identified variability in care and utilized interventions to standardize transition and transfer preparation across multiple domains and close practice gaps between IBD‐focused and general gastroenterology physicians.
- Standardizing transition and transfer across domains such as disease control/physical health, psychosocial well‐being, and transition and transfer planning/coordination has the potential to improve outcomes for AYAs during this clinically vulnerable time.

## 1 INTRODUCTION

Inflammatory bowel disease (IBD) is a chronic inflammatory condition of the gastrointestinal (GI) tract with a growing incidence around the world.^1^, ^2^ During adolescence or early adulthood, patients with pediatric‐onset IBD must transfer to the adult healthcare setting for ongoing management.^3^ The immediate post‐transfer period is known to be high‐risk, especially for adolescents and young adults (AYAs) with psychological disorders, and is associated with increased rates of poor disease control, lapses in routine care, increases in unplanned care, difficulty with adherence, and decreased quality of life.^4^, ^5^, ^6^, ^7^, ^8^, ^9^, ^10^ As such, efforts to improve healthcare transition, the coordinated, planned process over time to equip young adults with the knowledge and skills necessary to thrive in the adult setting, have been a focus across chronic diseases.^3^ A variety of transition interventions, including educational programs, joint pediatric‐adult clinics, and involvement of care navigators, have demonstrated improved patient outcomes, including symptom control, adherence, quality of life, and decreased health care utilization.^11^, ^12^ Despite this, transition practices for AYAs with IBD are heterogeneous and often absent.^13^

Understanding the high stakes and complexity of the transition and transfer process, we sought to use quality improvement (QI) methodology to measure and standardize the comprehensiveness of transition and transfer care provided to AYAs with IBD at our center. Specifically, we developed then utilized a QI tool called a Care Index which includes a list of prioritized variables to be completed for each patient to standardize care and reduce variability.^14^ Use of a Care Index has previously been shown to reduce variability and improve clinical outcomes across several chronic conditions, including pediatric cancer, lung transplantation, tracheostomy care, and in the management of children with celiac disease.^15^, ^16^, ^17^, ^18^, ^19^, ^20^, ^21^ Given the variation in care that exists among AYAs with IBD transitioning from pediatric to adult IBD care, we developed a Care Index focused upon standardizing each patient's preparation before transfer to adult IBD care known as the IBD Transition Care Index (TCI). The aim of this QI project was to increase the percentage of TCI variables completed among AYAs with IBD transferring to adult care from a baseline of 60% to greater than 80% by December of 2023 and sustain.

## 2 METHODS

### 2.1 Ethics statement

This project was reviewed by the Nationwide Children's Hospital Institutional Review Board (IRB) and deemed not to be human subjects research.

### 2.2 Local context

At the Center for Pediatric and Adolescent IBD at Nationwide Children's Hospital, 33 physicians follow over 900 patients with IBD. Patients are encouraged to attend a yearly IBD annual visit (IBD AV) where they are evaluated by the multidisciplinary IBD team. This visit takes the place of a regular visit with their primary GI physician and includes the following components: the IBD nurse practitioner (NP) performs a clinic visit, orders relevant medications and diagnostic studies, and addresses health maintenance topics; the psychologist completes psychosocial screeners and refers patients to appropriate care as needed; the dietitian performs a nutritional assessment and creates a personalized management plan; and the social worker assesses for barriers to care and provides resources related to insurance, school, and work accommodations. IBD AV appointments last approximately 90 min and can be completed in‐person or via telemedicine. Additional details of the IBD AV as well as role descriptions for the multidisciplinary team and data tracking processes at our IBD Center have been previously described.^22^

In AYA patients, the majority of transition preparation and transfer coordination are also delivered during the IBD AV. Patients are guided by multidisciplinary team members through a locally developed transition curriculum aimed at building the knowledge and self‐management skills necessary for transfer to adult care. Transition readiness is measured via a locally developed Transition Task Checklist, which assesses patients' self‐reported ability to complete a variety of important self‐management tasks (Supporting Information S1: Data 1). Checklist responses are reviewed by IBD AV clinicians and used to guide tailored goal setting and education based on skill deficiencies. Goals and educational interventions are documented by IBD AV clinicians in their notes. Selection of and referral to an appropriate adult GI provider commonly occurs during these visits as well. Despite these local procedures, there was no process in place to determine the consistency with which care was being delivered before transfer, including the rate of IBD AV attendance, the reliability with which patients saw each member of multidisciplinary team, the standardization of visit content, and the frequency with patients were being seen in the adult setting in a timely fashion. The identification of these gaps before transfer of care prompted our IBD team to develop the TCI.

### 2.3 Interventions

#### 2.3.1 Development of the IBD TCI

The TCI consisted of 10 variables that reflect a comprehensive, multidisciplinary approach to transition preparation and transfer to adult care. Variable selection was based on previously published recommendations for transition and transfer practices^10^, ^23^, ^24^, ^25^, ^26^, ^27^ and local expert consensus. The TCI was organized into three domains: 1. Disease Control/Physical Health, 2. Psychosocial Well‐being, and 3. Transition and Transfer Process (Table 1). Unless otherwise specified, each variable was expected to have been completed during the 12 months before or within the 3 months after the referral to adult care was placed.

**Table 1:** Transition Care Index domains, variables, and definitions.

Domains	Definition
Disease control/physical health	
Primary GI physician visit	Completed 12 months before or 3 months after referral
Dietitian visit	
Therapeutic drug monitoring^a^	
Mucosal healing assessment^b^	
Psychosocial well‐being	
Psychologist visit	Completed 12 months before or 3 months after referral
Social work visit	
Transition and transfer process	
IBD annual visit	Completed 12 months before or 3 months after referral
Transition task checklist ever complete^c^	Yes/no
Referral to adult GI provider placed	
Adult GI appointment attended	Completed within 6 months of referral

Abbreviations: GI, gastroenterology; IBD, inflammatory bowel disease.

^a^ If applicable.

^b^ Inclusive of completed colonoscopy or fecal calprotectin measurement.

^c^ Available starting Quarter 3 of 2020.

##### 2.3.1.1 Disease control/physical health

The disease control/physical health domain included four variables: 1. Having had a visit with one's primary GI physician, 2. Assessment by a dietitian, 3. Assessment of mucosal healing (either colonoscopy or fecal calprotectin), and 4. Therapeutic drug monitoring (TDM) for patients treated with medications for which TDM is available. While these variables are not traditionally included in transition preparation algorithms, they were included as a proxy for disease control, as prior studies show that transfer of care ideally occurs when a patient is in remission on a stable treatment regimen.^9^, ^24^

##### 2.3.1.2 Psychosocial well‐being

The psychosocial well‐being domain included two variables: 1. Assessment by a psychologist, and 2. Assessment by a social worker. Similar to the comment above, while these variables are not traditionally measured as components of transition readiness, they were chosen based on literature showing the significant psychosocial impacts of IBD and the fact that behavioral health diagnoses as well as social determinants of health have been predictors of poor transition outcomes in patients with IBD.^9^, ^28^

##### 2.3.1.3 Transition and transfer process

The transition and transfer process domain included four variables: 1. Attendance at an IBD AV. 2. Completion of the Transition Task Checklist (ever) to ensure at least one assessment of transition readiness before transfer, 3. Placement of a referral to adult GI, and 4. Confirmation of attendance at the initial adult GI visit (within 6 months of referral). The latter two variables were chosen to confirm that transfer of care was coordinated, complete medical records were sent to the adult GI provider, and that transfer to adult care was completed.

#### 2.3.2 Data collection

Patient data, including completion rates of TCI variables, were recorded in a secure electronic database. The reference date from which the timeliness of completion of TCI variables was calculated was the date the referral to adult care was placed in the electronic medical record (EMR). For patients who did not have a formal referral placed to an adult provider (i.e., self‐referred or lost to follow‐up), the date of the last clinic visit in the pediatric setting was used.

For patients who transferred care between January 2019 and December 2021 (pre‐intervention period), data were collected via retrospective chart review. During the post‐intervention period (January 2022 to June 2023), data were collected at the time an IBD team member was notified that a patient was planning to transfer care. If variables within the TCI were identified and incomplete at this time, recommendations were provided to their primary GI physician for completion before transfer.

#### 2.3.3 Key driver diagram (KDD) and interventions

**Figure 1:**
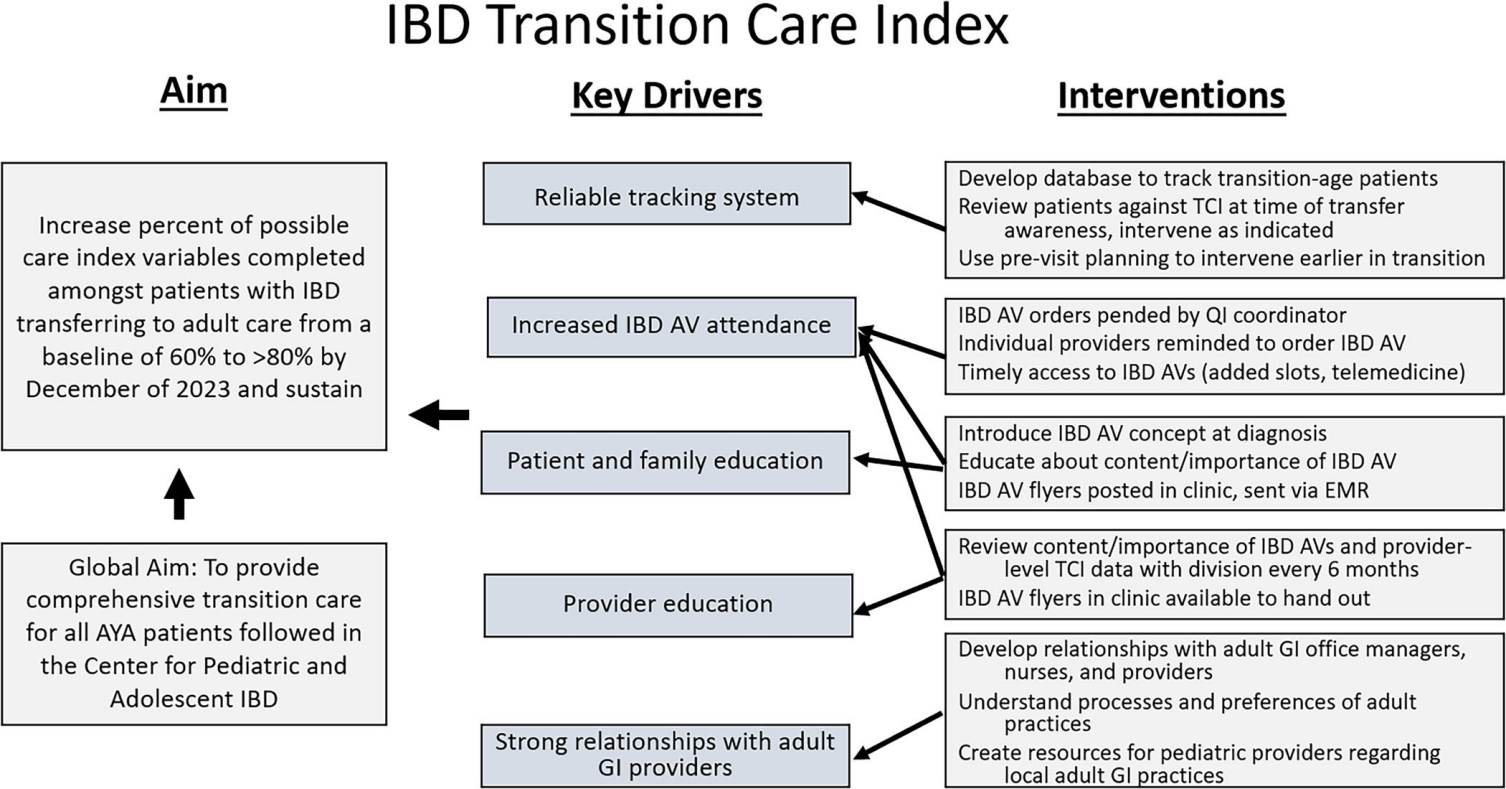
Key driver diagram for TCI project. AYA, adolescent and young adult; EMR, electronic medical record; GI, gastroenterology; IBD, inflammatory bowel disease; IBD AV, IBD annual visit; PVP, pre‐visit planning; QI, quality improvement; TCI, Transition Care Index.

A KDD was developed to summarize the project aim, primary drivers related to achieving this aim, and interventions targeted at improving completion of the TCI (Figure 1). A variety of interventions to increase referral rates to IBD AVs were initiated; some occurred before initiation of this project and continued throughout, including expanding access to IBD AVs by providing in‐person or telemedicine options and automatically pending referral orders for those in need of an IBD AV in the EMR. Several other interventions were rolled out at project onset; we developed educational flyers highlighting transition, transfer, and the value of IBD AVs, which were distributed to GI providers, patients, and caregivers at clinic visits and via the EMR. We also initiated transparent, physician‐level review of TCI data biannually to demonstrate differing practices and provide the opportunity for intervention. Finally, we reviewed lists of patients 18 years of age and older biannually to determine if an IBD AV was indicated and communicated this to their primary provider. To improve rates of Transition Task Checklist completion, it was built into our EMR and automatically sent to patients via the patient portal before visits. Incomplete Transition Task Checklists could also be completed in real time at the visit if needed. Results were recorded in the EMR and could be referenced at any time.

Finally, we strengthened relationships with local adult GI providers by scheduling virtual meetings, which allowed us to meet one another, identify a physician at each adult practice to serve as the transfer point‐person, and become familiar with services available at each adult practice. We developed a list of providers at each practice who were interested in seeing AYAs with IBD. These meetings and ongoing email communication allowed our IBD team to personalize the transfer process and ensure it occurred in a streamlined and timely fashion.

### 2.4 Assessments

The primary outcome was the percentage of TCI variables completed among AYAs who transferred to adult care. These data, and the frequency with which each individual TCI variable was completed, were displayed on p‐charts. Rates of variable completion were compared between two groups: IBD‐focused physicians (*N* = 4) and GI fellows (*N* = 9) (who are primarily precepted by IBD‐focused physicians in clinic) and general GI physicians (*N* = 20) (who care for patients with IBD but not as their primary role).

### 2.5 Analyses

Means and standard deviations were used for descriptive statistics. Frequencies of completion of TCI variables in the pre‐ and post‐periods between IBD‐focused and general GI physicians, as well as by patient age and sex, were compared using paired *t*‐tests.

## 3 RESULTS

### 3.1 Patient demographics

A total of 322 patients were transferred to adult care during the project period, 211 in the pre‐intervention period and 121 in the post‐intervention period (Table 2).

**Table 2:** Patient demographics.

Characteristic	Pre‐intervention	Post‐intervention	Total
Age at time of transfer, years, mean (SD)	20.7 (1.7)	21.1 (1.6)	20.9 (1.6)
Sex, *N* (%)			
Female	96 (45)	62 (51)	158 (48)
Male	115 (55)	58 (49)	173 (52)
Disease type, *N* (%)			
Crohn disease	159 (75)	85 (71)	244 (74)
Ulcerative colitis	42 (20)	30 (25)	72 (21)
IBD‐unclassified	10 (5)	5 (4)	15 (5)

Abbreviations: IBD, inflammatory bowel disease; SD, standard deviation.

### 3.2 TCI variable completion

**Figure 2:**
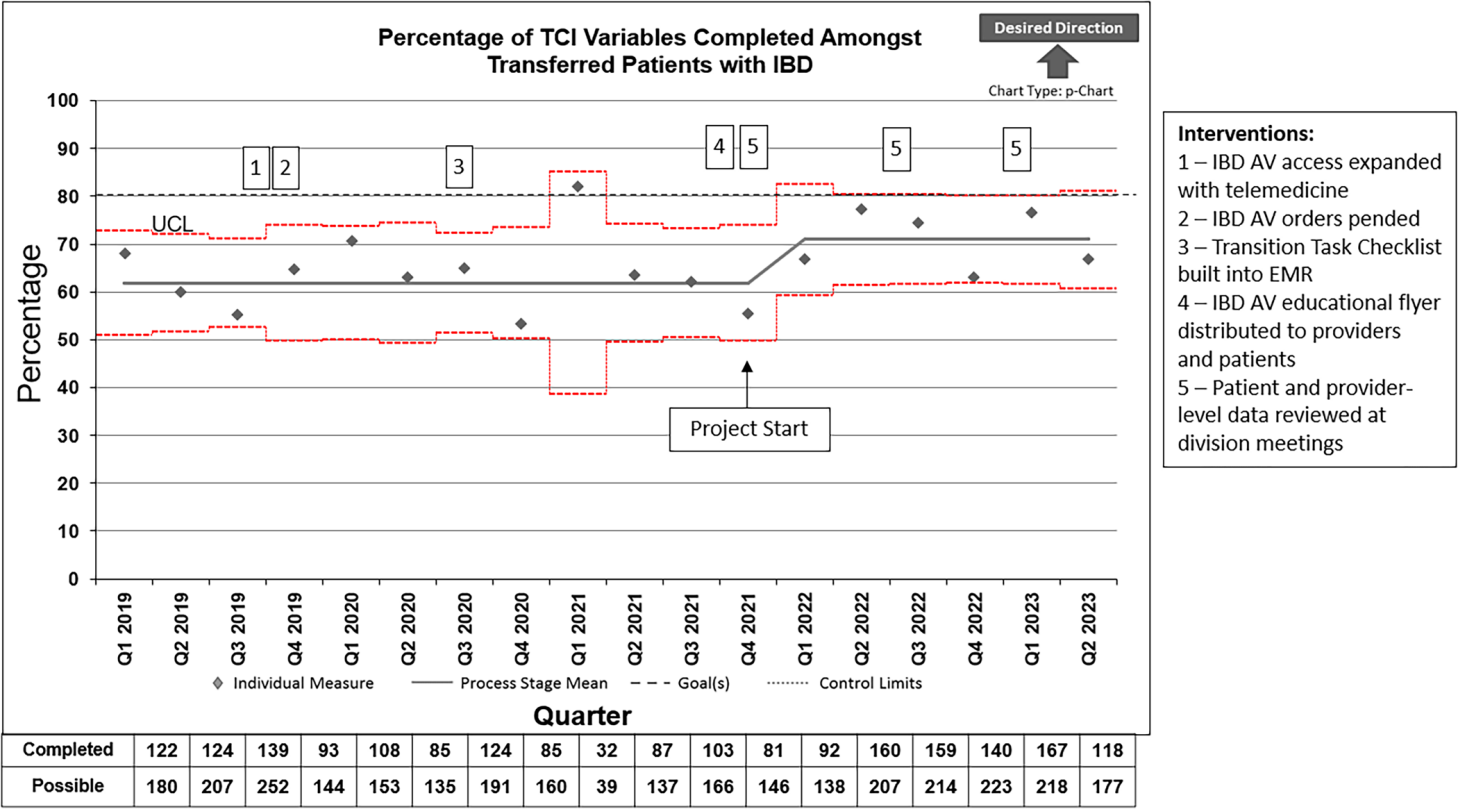
Percentage of TCI variables completed among transferred patients with IBD. EMR, electronic medical record; IBD, inflammatory bowel disease; IBD AV, IBD annual visit; TCI, Transition Care Index; UCL, upper confidence limit.

The total percentage of TCI variables completed increased after project implementation from a baseline of 62%–71% (Figure 2). There were no differences in TCI variable completion by sex or patient age (<20 vs. ≥20 years).

There were numerical improvements in completion rates of the following variables in the post‐ versus pre‐intervention period, though they did not reach statistical significance: visit with primary GI physician, psychologist, dietitian, or social worker, referral to adult GI provider placed, adult GI appointment attended, and completion of TDM (Table 3). There was a small, nonsignificant decrease in rates of mucosal healing assessment in the post‐ versus pre‐intervention period; of note, relatively low rates of mucosal healing assessment (approximately 60%) were driven by incomplete calprotectin completion as nearly 90% of patients had calprotectin completed *or ordered*. There was a significant increase in both the percentage of patients completing an IBD AV as well as Transition Task Checklist completion (Table 3). Similar to what was found with calprotectin completion described above, when we took into consideration the percentage of transferred patients who had had an IBD AV completed or *ordered* in the post‐intervention period, the rate improved to 81%.

**Table 3:** IBD variable completion rates pre‐ versus post‐intervention.

	Pre‐intervention	Post‐intervention	*p*
Disease control/physical health			
Primary GI physician visit	84%	88%	0.38
Dietitian visit	56%	56%	0.99
Therapeutic drug monitoring	77%	88%	0.09
Mucosal healing assessment	62%	58%	0.59
Psychosocial well‐being			
Psychologist visit	47%	62%	0.09
Social work visit	49%	54%	0.49
Transition and transfer process			
IBD annual visit	51%	62%	0.03*
Transition task checklist ever complete	37%	62%	0.006*
Referral to adult GI provider placed	88%	92%	0.257
Adult GI appointment attended	81%	88%	0.100

*Note*: *Indicate statistically significant with *p* value < 0.05.

Abbreviations: GI, gastroenterology; IBD, inflammatory bowel disease.

### 3.3 Comparison of TCI variable completion between IBD‐focused and general GI physicians

**Figure 3:**
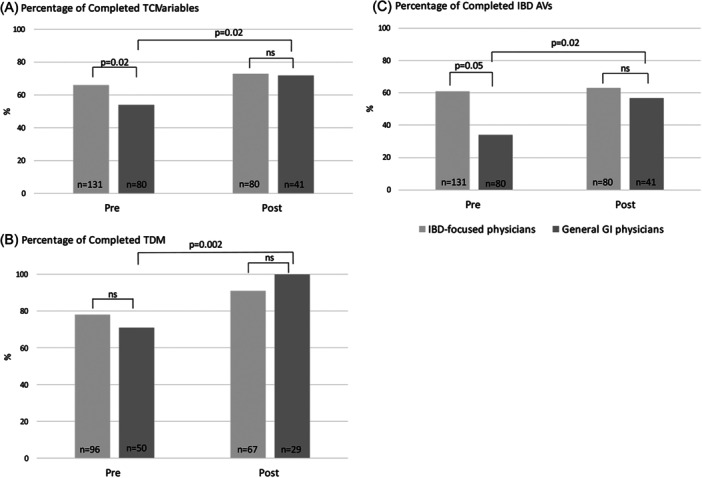
Comparison of completion of select TCI variables among IBD‐focused versus general GI physicians. (A) Total TCI variable completion; (B) TDM completion; (C) IBD annual visit completion. *p* < 0.05 is considered statistically significant. GI, gastroenterology; IBD, inflammatory bowel disease; ns, not significant; TCI, Transition Care Index; TDM, therapeutic drug monitoring.

When comparing rates of overall TCI variable completion for patients cared for by IBD‐focused versus general GI physicians, a significant pre‐intervention difference existed between these groups (66% vs 54%, *p* = 0.02). This difference resolved in the post‐intervention period (73% vs. 72%, *p* = 0.9; Figure 3A). Among general GI physicians, the percentage of TCI variables completed increased from a baseline of 54%–72% (*p* = 0.02) after project implementation. In addition, TDM completion rates increased among the general GI physicians between the pre‐ and post‐TCI periods from a baseline of 71%–100% (*p* = 0.002) (Figure 3B).

Finally, at baseline, a significant difference in IBD AV completion rates existed between IBD‐focused and general GI physicians (62% vs 34%; *p* = 0.05). IBD AV completion rates among general GI physicians increased from a baseline of 34%–57%, (*p* = 0.02) in the pre‐ versus post‐intervention period (Figure 3C). After project implementation, the completion of IBD AVs was similar for the groups.

## 4 DISCUSSION

The process of transition and transfer among young adults with chronic diseases like IBD is complex and often heterogeneous or missing entirely. Despite having a transition program delivered primarily through multidisciplinary IBD AVs at our IBD center, significant variability existed in terms of patient preparation before transfer. We used QI methodology to create the TCI, which aimed to measure and standardize not only transition preparation and transfer services delivered to young adults but also provision of medical and psychosocial services in an attempt to provide more holistic care before transfer. With a 72% TCI variable completion rate among young adult patients with IBD transferring to adult care, we fell short of our goal of 80%; however, we observed significantly decreased variability in practice between IBD‐focused and general GI physicians after project implementation.

The greatest impact of the TCI was its ability to identify differences in practice patterns between IBD‐focused and general GI physicians, providing the opportunity to introduce interventions to close these gaps. There was a nearly 20% increase in both overall TCI variable completion and completion of a multidisciplinary IBD AV among patients of general GI physicians following initiation of this project. As previously highlighted, at our IBD Center, the IBD AV provides patients the opportunity to receive a biopsychosocial model of care, address barriers to successful transition, and support individualized preparation for future transfer. Before project implementation, the IBD AV was being underutilized, especially among the general GI practitioners. Multiple interventions to improve IBD AV attendance were applied throughout the project, often simultaneously, making it difficult to identify which interventions were the most impactful. Our sense, however, is that regular, provider‐level data review allowed providers to be aware of their practice patterns and place referrals when indicated. Similar findings have been noted in studies describing the successful use of Care Indices to standardize cancer, transplant, and celiac care.^14^, ^15^, ^18^ In an era of superspecialization, where IBD care is becoming more complex and advanced IBD fellowships are becoming more common, measuring and reducing variability in care to patients by providers is essential.

Another unique aspect of the TCI was the inclusion of variables related to both disease control and psychosocial assessment. While these variables are not traditionally considered markers of transition readiness, prior literature has consistently found associations between both active disease and concomitant psychosocial issues and suboptimal outcomes after transfer of care.^9^, ^24^ Once again, this highlights the value of the IBD AV, where the IBD NP assesses disease activity and can order TDM, fecal calprotectin, or endoscopies if indicated, and the IBD psychologist competes a psychological evaluation and delivers targeted interventions to address common barriers to transition and transfer including mental health diagnoses and poor adherence.^9^ While we did not directly measure disease activity in this project, we tracked completion of both TDM and mucosal healing assessments before transfer to reduce the possibility of transferring at a time of unrecognized active disease or suboptimal medication dosing/adherence.^29^ Rates of TDM completion were high both before and after project initiation. Rates of completion of mucosal healing assessment were unchanged throughout the project, though we found nearly all patients had had a fecal calprotectin ordered, which they often did not complete. While not statistically significant, we also observed improved rates of psychological assessment in the post‐intervention period. This improvement was likely due to both increased participation in IBD AVs and a recent effort to integrate pediatric psychologists into routine IBD clinic visits when appropriate.^22^

Finally, improvement in the overall TCI completion rate was driven in part by increased rates of adult GI referral placement and confirmation of timely completion of the first adult GI visit. While these improvements were not statistically significant, we believe that placement of an official referral helps ensure the adult GI providers are aware of the upcoming transfer and have received complete medical records, a commonly cited barrier to transition and transfer by adult GI providers.^7^, ^30^ Timely completion of an adult GI visit helps to ensure continuous care receipt and avoid increases in unplanned emergency care for which post‐transfer patients are at increased risk.^31^ Development and strengthening of relationships with local adult GI providers was paramount to our ability to confidently refer patients and ensure they would receive excellent care.

Barriers to achieving our goal of 80% TCI variable completion among transferred patients were driven by the following factors. First, patients were offered but not required to attend IBD AVs. If they declined the referral due to personal preference or insurance denial of components of the visit, multiple TCI variables may have been missed. Second, patient adherence to ordered testing limited the completion of TCI variables. As mentioned previously, stool calprotectin was ordered in nearly all patients, but frequently not submitted. Finally, though the majority of patients were referred to IBD AVs, routine visits with their primary GI physician, and adult GI physicians, missed visits occurred and contributed to us not reaching our goal.

Our project is not without limitations. While we demonstrated improvement toward standardizing our transition process, future studies are needed to measure the impact of TCI completion upon outcomes such as remission rates, healthcare utilization, patient quality of life, and patient satisfaction after transfer to adult care. In this project, we sought to ensure patients had at least one assessment of transition readiness via the Transition Task Checklist before transfer of care. We acknowledge that ideally, repeated assessments of transition readiness are needed to track progress over time. Also, tracking a substantial number of variables manually can be burdensome. To ensure the sustainability of this study, a Care Index with fewer variables or automated data collection could be considered. An additional implementation barrier may include access to a multidisciplinary team. Centers would need to design a local TCI based on available local resources. Prior work on multidisciplinary IBD care suggests team members who may be able to serve certain roles on the team if other members are not available.^22^

## 5 CONCLUSION

In conclusion, transition and transfer are inevitable for young adults with IBD, and preparation is often variable, limited, or absent. QI methods, like the TCI, can be used to standardize expected care, track complex clinical processes, and reduce variability with the goal of improving patient outcomes.

## CONFLICT OF INTEREST STATEMENT

The authors declare no conflicts of interest.

## Supporting information


**Supplemental Data 1.** Transition Task Checklist.
