# Supplementary figures and images for: The Transition Care Index: Standardizing comprehensive transition and transfer for young adults with inflammatory bowel disease
Source: JPGN Rep. 2025 Jun 17;6(3):227–35. doi: 10.1002/jpr3.70045 (PMC12350045; doi:10.1002/jpr3.70045)

Supplemental Figure 1. Transition Task Checklist


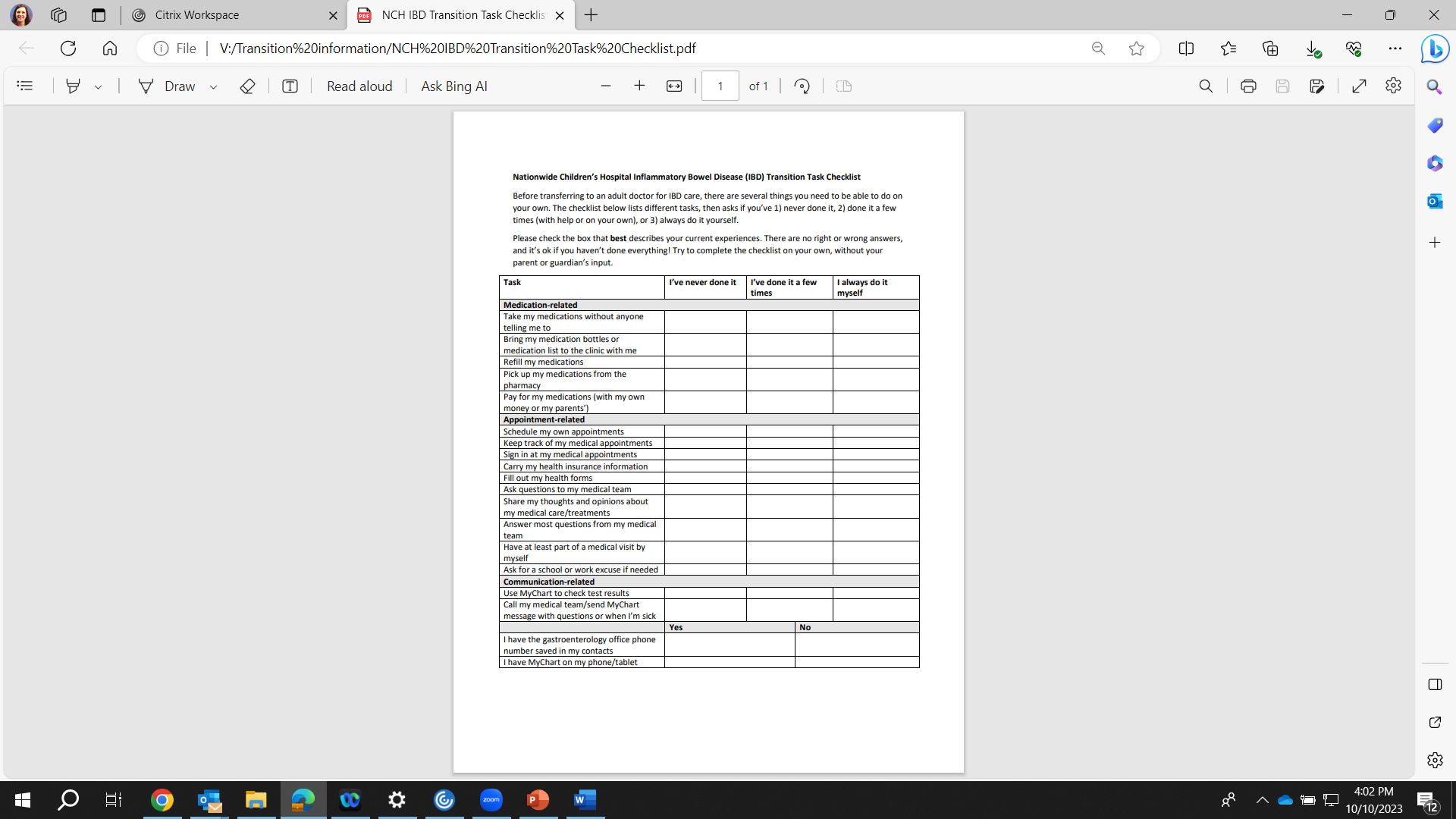

Supplement: Supplementary file 1 — Supplemental Data 1. Transition Task Checklist. [file JPR3-6-227-s001.docx]
